# Comparative Genomic Analysis of a Multidrug-Resistant *Staphylococcus hominis* ShoR14 Clinical Isolate from Terengganu, Malaysia, Led to the Discovery of Novel Mobile Genetic Elements

**DOI:** 10.3390/pathogens11121406

**Published:** 2022-11-23

**Authors:** Esra’a I. Al-Trad, Ainal Mardziah Che Hamzah, Suat Moi Puah, Kek Heng Chua, Stephen M. Kwong, Chew Chieng Yeo, Ching Hoong Chew

**Affiliations:** 1 Centre for Research in Infectious Diseases and Biotechnology (CeRIDB), Faculty of Medicine, Universiti Sultan Zainal Abidin, Kuala Terengganu 20400, Malaysia; 2 Faculty of Health Sciences, Universiti Sultan Zainal Abidin, Kuala Nerus 21300, Malaysia; 3 Department of Biomedical Science, Faculty of Medicine, University of Malaya, Kuala Lumpur 50603, Malaysia; 4 Infectious Diseases & Microbiology, School of Medicine, Western Sydney University, Campbelltown 2560, New South Wales, Australia

**Keywords:** *Staphylococcus hominis*, multidrug resistance, whole genome sequencing, genomic islands, plasmids, prophages

## Abstract

*Staphylococcus hominis* is a coagulase-negative *Staphylococcus* (CoNS) commensal capable of causing serious systemic infections in humans. The emergence of multidrug-resistant *S. hominis* strains is of concern but little is known about the characteristics of this organism, particularly from Malaysia. Here, we present the comparative genome analysis of *S. hominis* ShoR14, a multidrug-resistant, methicillin-resistant blood isolate from Terengganu, Malaysia. Genomic DNA of *S. hominis* ShoR14 was sequenced on the Illumina platform and assembled using Unicycler v0.4.8. ShoR14 belonged to sequence type (ST) 1 which is the most prevalent ST of the *S. hominis* subsp. *hominis*. Comparative genomic analysis with closely related strains in the database with complete genome sequences, led to the discovery of a novel variant of the staphylococcal chromosome cassette *mec* (SCC*mec*) type VIII element harboring the *mecA* methicillin-resistance gene in ShoR14 and its possible carriage of a SCC*fus* element that encodes the fusidic acid resistance gene (*fusC*). Up to seven possible ShoR14 plasmid contigs were identified, three of which harbored resistance genes for tetracycline (*tetK*), chloramphenicol (*catA7*), macrolides, lincosamides, and streptogramin B (*ermC*). Additionally, we report the discovery of a novel mercury-resistant transposon, Tn*7456*, other genomic islands, and prophages which make up the *S. hominis* mobilome.

## 1. Introduction

*Staphylococcus hominis* is a coagulase-negative *Staphylococcus* (CoNS) belonging to the phylum Firmicutes. Despite its commensal status, *S. hominis* is capable of causing infections particularly in immunocompromised patients where it has been reported to cause bacteremia, endocarditis, and endophthalmitis [1,2,3]. Multidrug-resistant (MDR) *S. hominis* strains have emerged [4,5], thus making the treatment of infections associated with this bacteria more challenging. *S. hominis* is also a repository for mobile genetic elements, such as the staphylococcal cassette chromosome *mec* element (SCC*mec*), which carries the *mecA* gene responsible for methicillin resistance [4,6]. Moreover, it contains plasmids that carry resistance determinants and to a lesser extent, virulence genes. Like their more well-renown, pathogenic, and drug-resistant cousin *Staphylococcus aureus*, CoNS strains which are resistant to macrolides, lincosamides, and streptogramins B (MLS_B_) antibiotics have emerged globally [7,8,9,10], and such resistance can either be the inducible MLS_B_ phenotype (iMLS_B_, which show resistance to macrolides and are susceptible to lincosamides but can be induced to lincosamide resistance) or the constitutive MLS_B_ phenotype (cMLS_B_, resistant towards macrolides, lincosamides, and streptogramin B).

Despite the clinical importance of CoNS such as *S. hominis*, there has been scarce information from Malaysia, particularly their genomic characteristics, where there have yet to be any published reports. Here, we present the draft genome sequence and analysis of *S. hominis* ShoR14, a multidrug-resistant, methicillin-resistant clinical isolate from Terengganu, Malaysia, to better understand its genome composition, and the molecular basis of its resistance and virulence mechanisms. We also present the results of comparative genome analysis of *S. hominis* ShoR14 which led to the discovery of several novel mobile genetic elements such as genomic islands, transposons, and a novel variant of SCC*mec* type VIII element. 

## 2. Results and Discussion

### 2.1. Phenotypic Antimicrobial Resistance Profile of S. hominis ShoR14

*S. hominis* ShoR14 was isolated as part of a routine hospital laboratory investigation. The in vitro antimicrobial susceptibility test demonstrated that ShoR14 belonged to the cMLS_B_ phenotype and was resistant to 10 antimicrobial classes which encompassed 14 antibiotics over 26 screened antibiotics, i.e., β-lactams (penicillin, oxacillin, and cefoxitin), fluoroquinolones (ciprofloxacin and intermediate resistance to moxifloxacin), macrolides (erythromycin), lincosamides (clindamycin), aminoglycosides (gentamicin), folate inhibitors (co-trimoxazole), fusidanes (fusidic acid), tetracyclines (tetracycline and intermediate resistance to doxycycline), phenicols (chloramphenicol), and monoxycarbolic acid (mupirocin). ShoR14 is thus classified as a multidrug-resistant (MDR) strain according to the criteria recommended by the joint commission of the U.S. Centers for Disease Control and Prevention (CDC) and the European Centre for Disease Prevention and Control (ECDC) [11]. This result was consistent with previous studies, which reported the emergence of multidrug- and methicillin-resistant *S. hominis* (MDR-MRSho) clinical isolates [4,5,6]. 

### 2.2. Genome Properties of Staphylococcus hominis ShoR14

The assembled draft genome of *S. hominis* ShoR14 (accession no. JAGHKT020000000) had 121 contigs, with a total length of 2,500,004 bp, *N*_50_ value of 156,448, and an average G+C content of 31.33%. The assembled genome contained 2523 protein-coding sequences (CDS), 51 transfer RNA (tRNA) genes, and 3 ribosomal RNA (rRNA) genes. Comprehensive genome analysis through PATRIC [12] characterized the subsystem distribution of genes of *S. hominis* ShoR14 with genes involved in metabolism (74 subsystems, 473 ORFs), protein processing (39 subsystems, 208 ORFs), energy (25 subsystems, 171 ORFs), and stress response, defense, and virulence (32 subsystems, 123 ORFs) being abundant.

### 2.3. Prediction of Antimicrobial Resistance and Virulence Genes from the S. hominis ShoR14 Genome Sequence

A search for potential antimicrobial resistance genes from the assembled *S. hominis* ShoR14 genome sequence led to the identification of the genes listed in Table 1. The discovered resistance genes revealing the genetic basis corresponding to its multidrug resistance phenotypic profile. 

Resistance towards tetracycline and doxycycline was likely conferred by the *tetK* gene that was located on a plasmid; this gene encoded an efflux pump that extrudes these antimicrobial agents [13,14]. Tetracycline resistance in staphylococci can be mediated by an efflux pump caused by the acquisition of the plasmid-encoded *tetK* and *tetL* genes [14,15] and/or due to ribosomal protection conferred by the *tetM* or *tetO* genes which are usually chromosomally located and often found in transposons [16,17]. It has been reported that strains carrying *tetK* were susceptible to minocycline, while strains carrying the *tetM* gene conferred resistance to all agents in the tetracycline class, including both tetracycline and minocycline [13,18,19]. The carriage of *tetK* in ShoR14 correlated to its phenotypic resistance profile which is resistance towards tetracycline, intermediate resistance towards doxycycline, and susceptibility to minocycline.

The reduced susceptibility of *S. hominis* ShoR14 to ciprofloxacin and moxifloxacin was possibly conferred by the *norA* gene that encodes a multidrug efflux pump [20]. Aminoglycoside resistance was likely due to the carriage of the *aac(6′)-aph(2″)* and *aadD* genes that encoded aminoglycoside acetyltransferase (AAC) and aminoglycoside adenylyltransferase (AAD) antibiotic inactivation enzymes, respectively. Additionally, resistance to penicillin was mediated by enzymatic antibiotic inactivation mechanisms via expression of the β-lactamase enzyme, which is encoded by the *blaZ* gene. Resistance towards oxacillin and cefoxitin (both are β-lactam antibiotics) was likely conferred by the *mecA* gene through a target alteration mechanism as the *mecA* gene encodes a modified penicillin-binding protein (PBP2a), which has low affinity towards methicillin and other β-lactam antibiotics [21]. Resistance to mupirocin and fusidic acid was mediated by the *mupA* and *fusC* genes, respectively, via a target alteration mechanism. Additionally, we found that the *fusC* gene was located in a SCC*mec* element, designated SCC*fusC*, which had been previously identified in *S. hominis* subsp. *hominis* and other *Staphylococcus* species [22]. Moreover, resistance towards erythromycin, clindamycin, and chloramphenicol was mediated by plasmid-encoded determinants as will be discussed later in this paper. Resistance to the co-trimoxazole, trimethoprim, and sulfamethoxazole antibiotics combination, was likely conferred by *sul4* and *dfrC* resistance genes via an antibiotic target replacement mechanism. The *sul4* gene, which encoded dihydropteroate synthase and confers sulfonamide resistance, was found to be widespread in Asia and Europe likely due to its occurrence on an integron and its association with an insertion sequence, IS*CR20* element [23], while *dfrC* encodes for dihydrofolate reductase (DHFR) which mediates trimethoprim resistance [24]. 

A search with the Virulence Factors Database (VFDB) led to the identification of several potential virulence genes which were categorized into three of the seven major staphylococcal virulence factor groups found in the VFDB (Table 2) (http://www.mgc.ac.cn/cgi-bin/VFs/genus.cgi?Genus=Staphylococcus accessed on 1 July 2022). A total of only 14 virulence genes were detected and a previous report had indicated that CoNS generally harbor lower numbers of virulence-associated genes when compared to *S. aureus* [2]. In stark contrast, the *S. hominis* strain Hudgins was reported to harbor 475 virulence factors out of a total of 2174 protein-coding genes [25]. The majority of these virulence factors are implicated in capsule biosynthesis, which plays a role in immune modulation/evasion by interfering with opsonophagocytosis [26]. Additionally, this strain carries two genes which are essential in adherence, i.e., *atl* gene that encodes for autolysin and the *ebp* gene that encodes for elastin binding protein, as well as *lip* and *nuc* genes which encode for lipase and thermonuclease exoenzyme, respectively.

### 2.4. In Silico Typing and Phylogenetic Analysis of S. hominis ShoR14

Six *S. hominis* housekeeping genes used in the MLST scheme were detected in the assembled *S. hominis* ShoR14 genome with the following alleles: *arc_6*, *glpk_5*, *gtr_7*, *pta_6*, *tpiA_6*, and *tuf_3*, classifying this isolate as sequence type 1 (ST1) [27]. *S. hominis* can be divided into two subspecies, i.e., *S. hominis* subsp. *Hominis* (*Shh*) and *S. hominis* subsp. *novobiosepticus* (*Shn*) that are difficult to differentiate phenotypically [27]. In a previous study, 40 STs in *S. hominis* were reported with three, i.e., ST2, ST16, and ST23, were *Shn*, while the remaining 37 STs were *Shh* with ST1 by far the most prominent type of this subspecies [27]. The ShoR14 isolate in the present study was ST1 and it is most likely *Shh.*

Although *S. hominis* ShoR14 harbored the *mecA* gene, the type of SCC*mec* element in which the gene was located was unable to be determined. Contradictory predictions were obtained using SCC*mec* finder whereby certain regions of either SCC*mec* type VIII(4A) or SCC*mec* type V(5C2 and 5) were identified over several contigs. Additionally, contig_38 of ShoR14 (accession no. JAGHKT020000038) also contained the *fusC* gene responsible for fusidic acid resistance, as mentioned earlier in Section 2.3. Interestingly, contig_38 shared larger regions of sequence identity to the SCC*fus* element as compared to either SCC*mec* types V(5C2 and 5) or VIII(4A) with 95% nucleotide sequence identity in the 2949 bp region that spanned the *ccrA1* recombinase gene and the two hypothetical ORFs that preceded it in SCC*fus* [nts. 14,511–17,463 of accession no. KF527883], and 99% sequence identity in the 1890 bp region that spanned the *fusC* gene [nts. 21,193–23,084 of KF527883] (Supplementary Figure S1). In contrast, contig_38 only shared 93% sequence identity over a smaller 712 bp region of either SCC*mec* type VIII(4A) or type V(5C2 & 5) which spanned part of a putative membrane protein. Mapping of the ShoR14 contigs to the SCC*fusC*, SCC*mec* type V(5C2 and 5), and SCC*mec* type VIII(4A) elements (Supplementary Figure S1) were inconclusive as there were contigs that were shared among all three elements (such as contig_22 and contig_33), and contigs that were exclusive for each of the SCC elements. It is therefore likely that *S. hominis* ShoR14 contained a novel SCC*mec* and/or SCC*fus* element(s) but in the absence of its complete genome sequence, it would be difficult for us to determine for certain the complete genetic structure(s). However, a subsequent comparison with the complete genome of *S. hominis* FDAARGOS_136 led to the discovery of a novel variant of a SCC*mec* type VIII element for which the SCC*mec* of ShoR14 shared extensive sequence similarity (see following Section 2.5). A previous study had reported that 15 % (5/34 isolates) of *S. hominis* clinical isolates harbored SCC*mec* type VIII(4A), although that study had derived its conclusion from the PCR results of certain conserved regions of the SCC*mec* element [6]. A more recent study of *S. hominis* and *S. haemolyticus* isolates from dogs showed the presence of novel SCC*mec* composite islands, all of which were initially categorized as non-typeable SCC*mec* (NT-SCC*mec*) [28]. 

**Figure 1:**
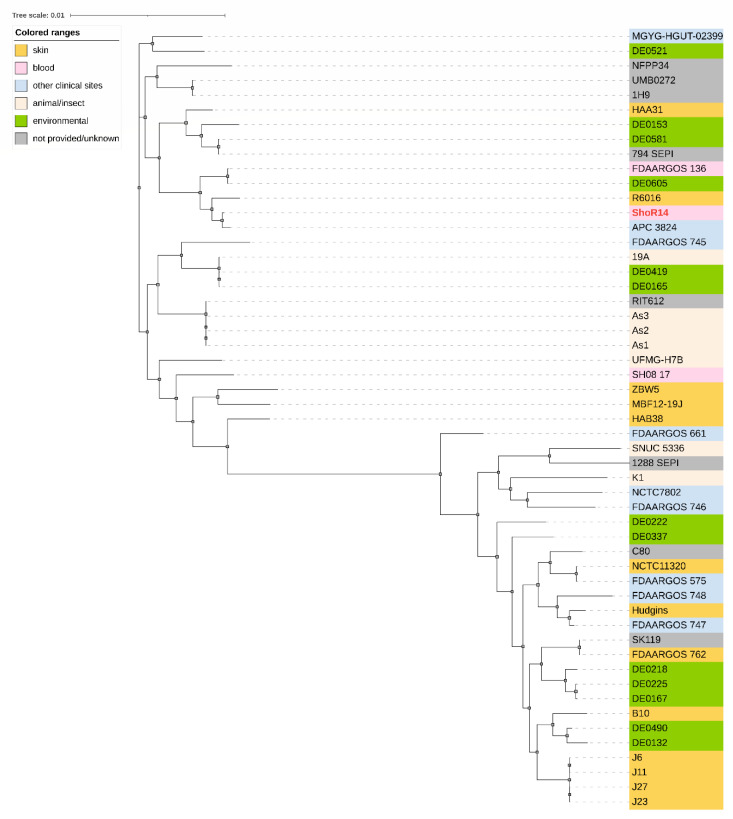
Core genome maximum-likelihood phylogenetic tree of *S. hominis* ShoR14 in comparison with other related *S. hominis* isolates (Supplementary Table S1). The *S. hominis* core genome comprises 1217 core genes from a total of 8679 genes. The source of the *S. hominis* isolates is depicted as colored labels and indicated on the upper left of the figure.

The core genome phylogenetic tree of *S. hominis* ShoR14 in comparison with other 52 *S. hominis* genomes in GenBank (Figure 1) showed that ShoR14 is most closely related to the *S. hominis* strain APC 3824 (accession number NZ_SHFC00000000.1) which was isolated from a human milk sample and is also ST1.

### 2.5. Comparative Genomic Analysis, Prediction, and Identification of Genomic Islands

**Figure 2:**
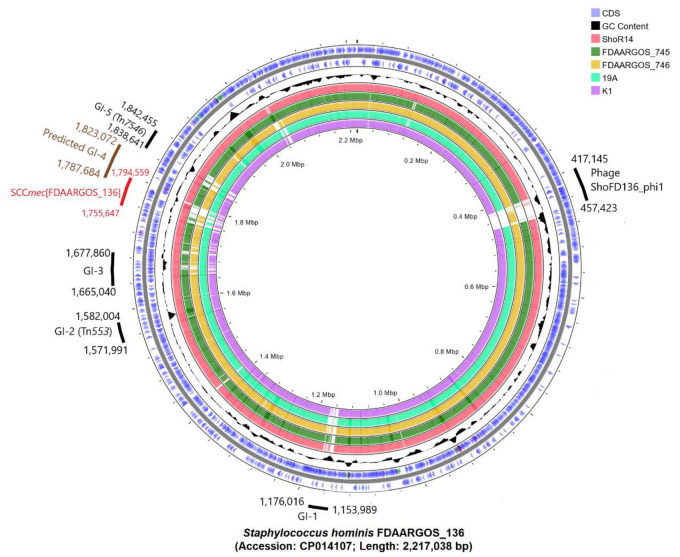
Comparison of the assembled genome sequence of *S. hominis* ShoR14 (accession no. JAGHKT020000000) using CGView with five reference *S. hominis* strains with complete genome sequences, i.e., *S. hominis* FDAARGOS_136, *S. hominis* FDAARGOS_745, *S. hominis* FDAARGOS_746, *S. hominis* 19A, and *S. hominis* K1. The *S. hominis* FDAARGOS_136 genome was used as the main reference genome for the CGView BLASTN comparison. From outside to center, rings 1 and 2 show protein-coding genes on both the forward and reverse strand of the FDAARGOS_136 genome; ring 3 shows the GC content of the FDAARGOS_136 genome; ring 4 shows the ShoR14 genome; ring 5 shows the genome of FDAARGOS_745; ring 6 shows FDAARGOS_746; ring 7 shows 19A; ring 8 (the innermost ring) shows the K1 genome. Genomic islands, the prophage, and the novel SCC*mec* (shown in red) found in the FDAARGOS_136 genome are labelled along with their nucleotide coordinates. The extent of genomic island 4 (GI-4) that was predicted by IslandViewer 4 was similarly indicated but in brown. This was eventually shown to be part of SCC*mec*[FDAARGOS_136] (see main text).

The draft genome sequence of *S. hominis* ShoR14 was compared with the complete genome sequences of *S. hominis* FDAARGOS_136 (accession no. CP014107, 2,217,038 bp), *S. hominis* FDAARGOS_745 (accession no. CP050982, 2,338,248 bp), *S. hominis* FDAARGOS_746 (accession no. CP046306, 2,323,613 bp), *S. hominis* 19A (accession no. CP031277, 2,202,898 bp), and *S. hominis* K1 (accession no. CP020618, 2,253,412 bp) using CGView. The comparison showed extensive synteny between the ShoR14 genome and the genomes of the above-mentioned reference strains. As the only draft genome sequence for ShoR14 is currently available, potential genomic islands were predicted using IslandViewer 4 on the complete genome of *S. hominis* FDAARGOS_136, which is the most closely related strain to ShoR14 with a complete genome sequence (as indicated in the phylogenetic tree in Figure 1). The average nucleotide identity (ANI) value between ShoR14 and FDAARGOS_136 was 99.66%. Five genomic islands, designated GI-1 to GI-5, were predicted by IslandViewer 4 based on the FDAARGOS_136 complete genome sequence (Figure 2 and Supplementary Table S2).

**Figure 3:**
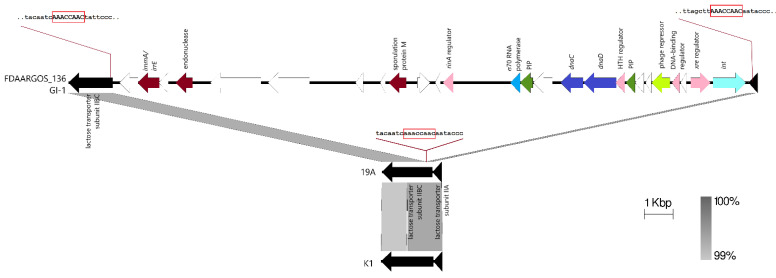
Genetic organization of the GI-1 island in the genome of *S. hominis* FDAARGOS_136. The 22,028 bp GI-1 was inserted at the 5’ end of the lactose transporter subunit IIBC gene in the FDAARGOS_136 genome, leading to an 8 bp direct repeat of the target sequence, AACCAAC, which was indicated within a red box. The sequences of the lactose transporter subunit IIBC gene at the point of insertion of GI-1 are shown for FDAARGOS_136, and the corresponding uninterrupted subunit IIBC gene in *S. hominis* 19A and *S. hominis* K1. The GI-1-encoded integrase (*int*) is depicted as a light blue arrow; pink arrows are for transcriptional regulators; dark blue arrows are DNA replication genes; blue arrow is the gene encoding for a σ^70^-type RNA polymerase; dark green arrows are pathogenicity island proteins (labelled here as PIP); lime green arrow is a gene encoding phage repressor protein; dark maroon arrows depict genes that have known homologs; and white arrows are open reading frames encoding hypothetical proteins. The extent of nucleotide sequence identities is shown as grey shaded areas, as depicted at the bottom right side of the figure. The linear maps here depict nts. 1,152,465–1,176,252 of *S. hominis* FDAARGOS_136 (accession no. CP014107), nts. 675,819–677,890 of *S. hominis* 19A (accession no. CP031277), and nts. 1,769,164–1,771,292 of *S. hominis* K1 (accession no. CP020618).

GI-1 was predicted by IslandViewer 4 to be 53,623 bp in length, spanning nts. 1,151,037–1,204,660 of the FDAARGOS_136 genome (Supplementary Table S2) but a closer scrutiny of the sequences and comparison with the other reference *S. hominis* genomes indicated that this island may be smaller than predicted. Most of the observed differences (presence or absence of genes) in this predicted island is in a region of ~22 kb in length that ranged from the hypothetical protein (locus tag: AL495_06095) encoded in nts. 1,154,231–1,154,872 of FDAARGOS_136 to the integrase (locus tag: AL495_06220) encoded from nts. 1,174,794–1,175,930. Aside from the integrase, this region also encodes several genes that are signature to staphylococcal genomic/pathogenicity islands [29] such as genes encoding DNA replication proteins, i.e., DnaC (AL495_06170) and DnaD (AL495_06175), phage-like proteins (AL495_06135 and AL495_06200), and pathogenicity island proteins (AL495_06160 and AL495_06185). This region is also flanked by a gene encoding a lactose transporter subunit IIBC (AL495_06090; nts. 1,152,465–1,154,000) and a gene encoding a lactose transporter subunit IIA (AL495_06230; nts. 1,176,258–1,176,572), which appeared as though the island had inserted itself in between these two genes, which are usually contiguous. Indeed, a comparison of similar regions in the *S. hominis* 19A and K1 genomes enabled the precise delineation of the boundaries for GI-1 which is 22,028 bp in length (spanning nts. 1,153,989–1,176,016 of the FDAARGOS_136 genome) and had inserted at the 5’ end of the lactose transporter IIBC gene, leading to an 8 bp direct repeat sequence of AAACCAAC (Figure 3). GI-1 appeared to be unique to *S. hominis* FDAARGOS_136 and was absent in *S. hominis* ShoR14 and other staphylococci genomes in the current database.

Genomic island 2 (GI-2), predicted by IslandFinder 4 as 10,013 bp in length, was identified in FDAARGOS_136, carrying eleven CDS (Supplementary Table S2), all of which were present in ShoR14 (contig_5) and the other reference *S. hominis* strains (Figure 2). Closer analysis of this island showed that it is actually Tn*553*, a 9050 bp transposon that carried the complete *blaZ-blaR1-blaI* β-lactamase operon initially discovered in *S. aureus* QD-CD9 integrated within the chromosomal *yolD* gene [29,30]. The full copy of Tn*553* was found in *S. hominis* ShoR14 [nts. 66,306–75,359 of contig_5 (accession no. JAGHKT020000005.1)]. Interestingly, in *S. hominis* FDAARGOS_136 and ShoR14, the site of integration for Tn*553* was identical. Tn*553* was reported to insert into target sites without producing direct repeats characteristic of most transposons but with differing 6 bp sequences at the left and right junctions of the transposon [29,30]. In both FDAARGOS_136 and ShoR14, these 6 bp sequences were identical to those of Tn*553* in *S. aureus* QD-CD9 (i.e., CAAAAG for the left junction, and TAAATG for the right junction). 

Genomic island 3 (GI-3) was predicted by IslandViewer 4 to be 11,216 bp in length in the FDAARGOS_136 genome with 14 CDS (spanning AL495_08620 which encodes a YitT family protein, to AL495_08685 which encodes a hypothetical protein) (Supplementary Table S2) and this structure was conserved in *S. hominis* 19A and ShoR14 (Figure 2) although in ShoR14, the putative GI-3 spanned three contigs, i.e., contig_26, contig_88, and contig_35. However, a closer examination of the genetic environment and comparisons with the genomes of the other reference *S. hominis* strains indicated the possibility of this putative genomic island being larger than predicted. The gene encoding the YitT family protein is flanked by a complete copy of the 789 bp IS*257* downstream (within the IslandViewer-predicted region) and a partial copy of IS*257* (586 bp) immediately upstream (which was not predicted by IslandViewer). Upstream of this partial copy of IS*257* is a CDS (AL495_08610) that was annotated as a resolvase with a serine recombinase domain. These two CDS are absent in the genomes of FDAARGOS_745, FDAARGOS_746, and K1 (Figure 2 and Supplementary Figure S2) and could possibly be part of this putative genomic island. Interestingly, further upstream is a 384 bp gene (AL495_08595) that encodes a putative mobilization protein with a MobC domain; *mobC* is usually plasmid encoded and the encoded protein is a relaxase that functions to mobilize plasmids (usually along with MobA and MobB proteins) at their origin of transfer, *oriT* [31]. This putative *mobC* is present in ShoR14 and K19 but is absent in FDAARGOS_745, FDAARGOS_746, and K1 (Supplementary Figure S2). Intriguingly, when this extended GI-3 region was compared using BLASTN, regions of similarity with several staphylococcal plasmids were observed. Notably, an approximately 6.1 kb region that spanned the *uspA* universal stress protein-encoding gene to the resolvase/recombinase gene and encompassing the partial and complete IS*257* copies showed >95% sequence identity with plasmid_1 carried by *Staphylococcus epidermidis* ATCC 14990 (Supplementary Figure S2), while a smaller 3.5 kb region spanning *uspA* to the partial copy of IS*257* showed >95% sequence identity with plasmid_2 of *S. hominis* FDAARGOS_746 and plasmid pSE459_1 of *S. epidermidis* SE459 (Supplementary Figure S2). However, no signature transposon-like sequences (such as terminal inverted repeats and/or direct repeat of target sequences) were detected within either of these 6.1 kb or 3.5 kb regions. Thus, unlike GI-1, we were unable to determine the precise borders of this putative GI-3 island as comparisons with other complete genomes or plasmids did not reveal signature transposon or island-like motifs. Moreover, no known phage-related or pathogenicity-island-related genes could be found within this putative GI-3 region. 

**Figure 4:**
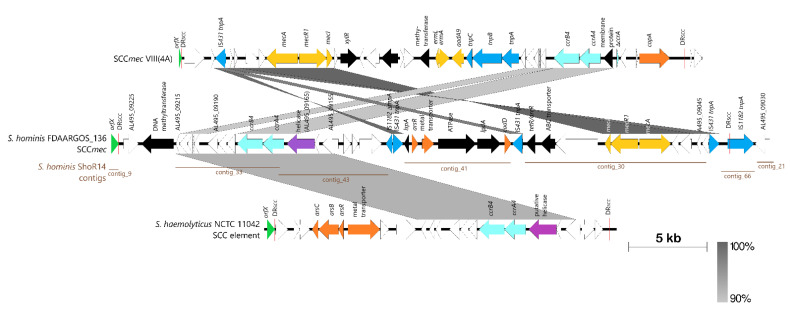
Comparative linear maps of the *S. hominis* FDAARGOS_136-encoded SCC*mec*[FDAARGOS_136] with SCC*mec* type VIII(4A) from *S. aureus* (accession no. FJ390057) and the SCC element from *S. haemolyticus* NCTC11042 (accession no. AB505631). The assembled contigs of *S. hominis* ShoR14 (accession no. JAGHKT020000000) were also mapped to SCC*mec*[FDAARGOS_136] and contigs with sequence identities of >90% were indicated as horizontal brown labeled lines below the SCC*mec*[FDAARGOS_136] linear map. The *orfX* which marks the beginning of the SCC element is shown as a green-filled arrow/triangle in each linear map, while the direct repeats that flank each SCC element are indicated as a red vertical bar labeled as “DR_SCC_”. Antibiotic resistance genes are shown as golden colored arrows; heavy metal resistance genes are depicted as orange arrows; sky blue arrows are the SCC recombinases; darker blue arrows are transposases; purple arrows indicate putative helicase; black arrows are CDS with known function or domains; and white arrows are CDS-encoding hypothetical proteins (for which some of the locus tags for those encoded by FDAARGOS_136 are shown). The extent of nucleotide sequence identities of >90% is shown as grey shaded areas with higher identities shown as darker shades of grey as depicted at the bottom right side of the Figure.

IslandViewer 4 predicted the fourth genomic island in FDAARGOS_136, GI-4, to span 31 CDS from AL495_09185 to AL495_09345 (about 35 kb in length) (Supplementary Table S2). Comparison of this region with other *S. hominis* genomes showed that almost 80% of this region from AL495_09230 to AL495_09345 (about 28.5 kb in length) was highly conserved and thus, not likely to be in a genomic island-like structure. However, CGView showed that the region from AL495_09225 to AL495_09035 (which was an additional 33.6 kb region upstream of the IslandViewer-predicted region) was highly variable (Figure 2) and this region included the *mecA-mecR1-mecI* genes that are central to a SCC*mec* element. SCCFinder was unable to determine the type of SCC*mec* element in the FDAARGOS_136 genome with the closest match being either SCC*mec* type VIII(4A) or SCC*mec* type V (5 and 5C2). To delineate the SCC*mec* element in FDAARGOS_136, we used the translated *orfX* sequence of SCC*mec* V (5 and 5C2) (accession no. BAK53093) in a TBLASTN search which led to the discovery of AL495_09230 as the most likely *orfX* of FDAARGOS_136 with 94% amino acid sequence identity. We next searched for the direct repeat sequences that are characteristically located at the borders of the SCC*mec* element that resulted from SCC*mec* insertion into the staphylococcal chromosome [32,33]. An 11 bp sequence at the 3’ end of *orfX* (CCACAAATGAT) was repeated 38,913 bp upstream within a gene encoding for an IS*1182* family transposase (AL495_09035), thereby marking the boundaries of this SCC*mec* element, which spans nts. 1,755,647–1,794,559 of the *S. hominis* FDAARGOS_136 genome. Since the International Working Group on the Classification of Staphylococcal Cassette Chromosome (IWG-SCC) no longer annotates or assigns new SCC*mec* subtypes to other species than *S. aureus* (Uehera, 2022), we thus designate this element as SCC*mec*[FDAARGOS_136], as suggested by the IWG-SCC. Analysis of the characteristic SCC*mec* genes in SCC*mec*[FDAARGOS_136] showed that it contains the class A *mec* complex (with 100% sequence identity to the class A *mec* complex in SCC*mec* type VIII) and a *ccrAB4* complex (with 90% identity to the *ccrAB4* complex carried in SCC*mec* type VIII). Thus, it can be concluded that SCC*mec*[FDAARGOS_136] is a variant or subtype of SCC*mec* type VIII and a comparative map is shown in Figure 4. 

Interestingly, mapping of the *S. hominis* ShoR14 contigs to SCC*mec*[FDAARGOS_136] showed extensive regions of sequence similarities with the SCC*mec* structure between the flanking direct repeats covered by five ShoR14 contigs (i.e., contig_30, contig_33, contig_41, contig_43, and contig_66) making up about 35.2 kb of the 38.9 kb SCC*mec*[FDAARGOS_136] sequence (or 90.5%) and with contig_9 covering the *orfX* gene along with the SCC direct repeat (Figure 4). Only a 3.7 kb region containing two CDS, AL495_09225 and AL495_09220 (a putative DNA methyltransferase), were not found in any of the ShoR14 contigs. Thus, it is very likely that *S. hominis* ShoR14 harbors a SCC*mec* type VIII variant that is similar to SCC*mec*[FDAARGOS_136]. Nevertheless, in the absence of a complete genome sequence for ShoR14, it is difficult to ascertain the final structure of its SCC*mec* and whether the *fusC* fucidin-resistance gene is present within the SCC*mec* or in a separate SCC*fus*-like element. Mapping of the ShoR14 contigs appeared to suggest the latter as contig_38 of ShoR14 which contained the *fusC* as well as the *ccrA1* gene which did not map onto SCC*mec*[FDAARGOS_136] but to SCC*fus* instead (see Section 2.4 and Supplementary Figure S1).

A closer look at the IslandViewer-predicted GI-5 led to the discovery of a novel transposon-like element, designated Tn*7546* by the Transposon Registry [34] which is 3789 bp and comprises the *merA*-encoded mercury reductase and its corresponding regulatory gene *merR*, and a transposase of the IS*L3* family, *tnpA*_ISL3_ (spanning nts. 1,838,641–1,842,445 of the FDAARGOS_136 genome). Tn*7546* is flanked by an 11 bp inverted repeat, GGGTCTTCGGA, and its insertion led to an 8 bp direct repeat of the target site sequence, AAAATAAG. This transposon is found in several other *S. hominis* strains including *S. hominis* ShoR14 (in contig_9) and 19A, as well as in a wide range of other Gram-positive bacteria (Table 3) where the 8 bp target site duplication was almost always identified (except for *Lysinibacillus fusiformis* NEB1292, where a 7 bp target site duplication was observed). The size of the transposon varied slightly from 3775–3791 bp with the nucleotide difference being in the non-coding regions of the transposon. Interestingly, the only description of Tn*7546* was in the genome of *Staphylococcus epidermidis* NW32 which harbored an 83.6 kb composite SCC island designated CI_32_ that consisted of three SCC elements [35]. In *S. epidermidis* NW32, Tn*7546* was part of a 24.3 kb SCC element designated SCC*_mer/pbp4/pts_* that contained a penicillin-binding protein 4 (PBP4)-encoding gene along with genes that encode sorbitol-related metabolism, as well as the *ccrA2* and *ccrB2* genes encoding the SCC cassette recombinases. Xue et al. (2017) did not, however, describe the *merR-merA-tnpA*_ISL3_ genes to be within a transposon-like structure [35]. 

### 2.6. Identification of S. hominis Prophages

**Figure 5:**
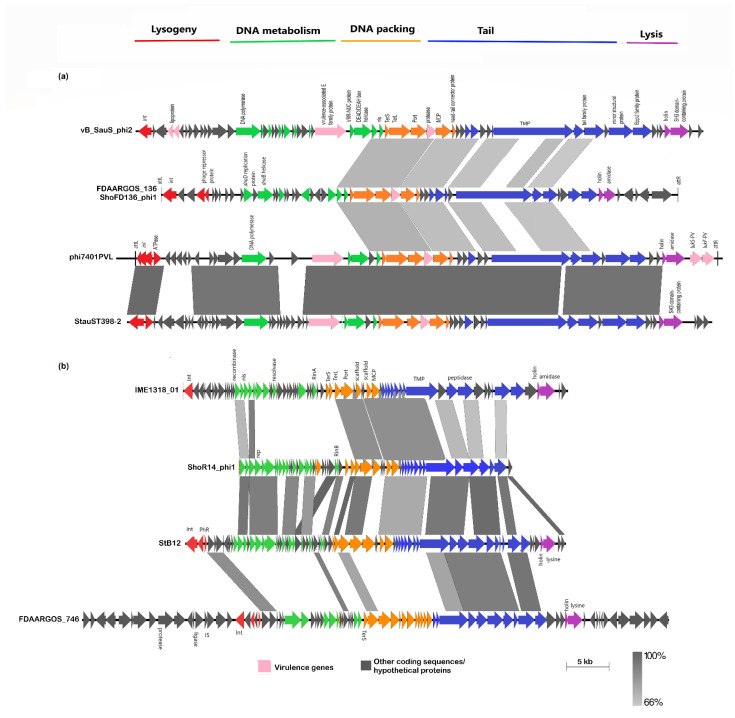
Comparative analysis of the ShoFD136_phi1 phage from *S. hominis* FDAARGOS_136 (**a**) and the ShoR14_phi1 phage from *S. hominis* ShoR1 (**b**) with related phage genomes. Functional phage modules are represented in different colors: lysogeny in red, DNA metabolism in green, DNA packaging in orange, phage tail in dark blue, and lysis in purple. Virulence genes are depicted in pink while other coding sequences and/or hypothetical proteins are shown in grey. Grey-shaded areas in between the linear maps indicate regions of nucleotide sequence identities of >66% with darker shades of grey depicting higher sequence identities as shown in the vertical bar at the bottom right side of the figure. Accession numbers of the phage genomes used in the comparative maps are as follows: vB_SauS_phi2 (accession no. NC_028862), phi7401PVL (accession no. NC_020199), StauST398-2 (accession no. NC_021323), IME1318_01 (accession no. KY653116), StB12 (accession no. NC_020490.2), and FDAARGOS_746 (accession no. CP046306, positioned at nts. 336,947–405,905). Abbreviations: Int, integrase; PhR, phage repressor; Rep, replication; nls, endonuclease; RinA and RinB, transcriptional regulator; TerS, terminase small subunit; TerL, terminase large subunit; Port, portal protein; MHP, major head protein; MCP, major capsid protein; MTP, major tail protein; TMP, tail measure protein.

Analysis of the CGView results (Figure 2) also led to the discovery of a novel prophage in the genome of *S. hominis* FDAARGOS_136 that was absent in ShoR14 and the other reference strains. PHASTER validated the presence of this phage in the genome of FDAARGOS_136. This 40,278 bp phage (spanning nts. 417,145–457,423 of FDAARGOS_136), which we designated ShoFD136_phi1, did not have any close homologs in the databases and shared only 77% sequence identity over slightly less than 10% coverage with the *S. aureus* phage vB_SauS-phi2 (KT186243) [36], and 76% sequence identity over 10.6% coverage with the *S. aureus* phage phi7401PVL [37], both of which were classified under the Siphoviridae family (Figure 5A). 

On the other hand, analysis of the ShoR14 genome with PHASTER revealed the presence of a putative prophage that was absent in FDAARGOS_136 but with some of its regions aligned with an intact phage approximately 68 kb in size in *S. hominis* FDAARGOS_746 (Figure 5B). The ShoR14-predicted phage, which we designate ShoR14_phi1 (~34 kb in length, and spanning multiple contigs), is more closely related to the *S. hominis* phage StB12 (accession no. NC_020490) that belongs to the *Siphoviridae* family (class II phage) and with a genome size of 44,714 bp [38,39]. Since the ShoR14_phi1 phage sequences were spread out over more than 20 contigs, we used MeDuSa (multi-draft-based scaffolder) [40] to construct a scaffold consensus sequence for the putative phage to enable comparison with StB12 and other related phages including the 45,236 bp *Siphoviridae* phage IME1318_01 from *Staphylococcus caprae* (accession no. KY653116) [41] and the 68 kb phage from FDAARGOS_746 (Figure 5B). Comparison of the scaffold sequences for the ShoR14_phi1 phage showed that they were missing two essential components, namely the lysogeny region (particularly the integrase gene) and the lysis region (exemplified by the holin and amidase genes) (Figure 5B). This meant that ShoR14_phi1 could be a remnant of a Stb12-like prophage that had lost the lysogenic and lysis genes, or that the sequences themselves could be lost in the assembly of the short reads. Nevertheless, ShoR14_phi1 is likely a novel *Siphoviridae* phage with its DNA metabolism and tail morphogenesis genes more related to Stb12, while its DNA packing and part of the tail morphogenesis genes were more related to phage IME1318_01 (Figure 5B).

### 2.7. Identification of Putative Plasmid Sequences in S. hominis ShoR14

Seven contigs from the assembled genome of *S. hominis* ShoR14 were found to harbor plasmid replicase genes indicating that they possibly originated from plasmids. BLASTN analysis of these contigs showed that they have plasmid counterparts in the databases with four of the contigs, namely contig_121 (21,512 bp), contig_46 (4439 bp), contig_49 (3836 bp), and contig_59 (2463 bp), likely complete plasmids (as they displayed >90% sequence identity and are identical or almost identical in size to their database plasmid counterparts). PCR using outward-directing primers was used to validate the size and sequences of these putative plasmids. For contig_58 which had an initial assembled size of 1759 bp, PCR followed by Sanger sequencing of the amplified product extended the size of the contig to 2508 bp. However, in the case of contig_27 (16,214 bp) and contig_53 (3025 bp), PCR using outward-directing primers did not lead to any amplified products inferring the likelihood that these are partial plasmid sequences. BLASTN comparison of these two contigs with other plasmids in the database was similarly inconclusive. 

**Figure 6:**
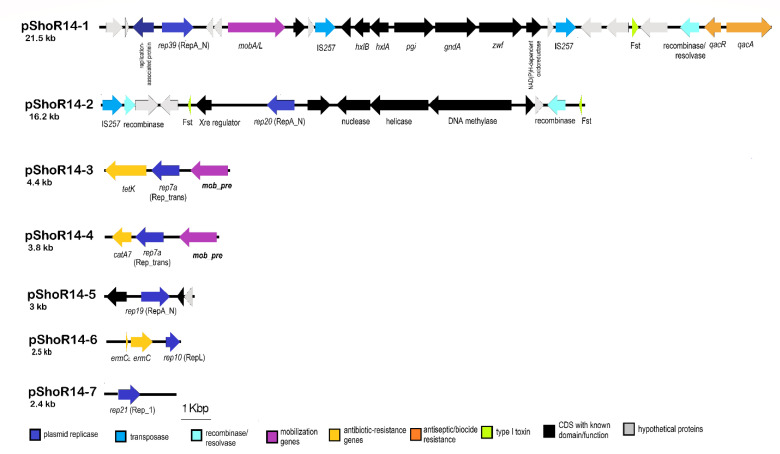
Linear maps of putative plasmids detected in the assembled genome sequence of *S. hominis* ShoR14. The types of CDS/genes that are found in these plasmid sequences are indicated in colored arrows and color-coded as labeled below the figure. Abbreviations: *rep*, replication initiator protein (replicase)-encoded gene; *ermC*, ribosomal RNA adenine N-6-methyltransferase gene that confers resistance to macrolides, lincosamides, and streptogramin B; *ermC_L_*, leader peptide of *ermC*; *catA7*, gene encoding chloramphenicol acetyl transferase; *tetK*, tetracycline resistance gene; m*ob_pre*, mobilization/recombination gene; Fst, toxin-antitoxin type I Fst toxin; *qacA*/*qacR*, biocide resistance genes; *hxlA*, 3-hexulose-6-phosphate synthase; *hxlB*, 6-phospho-3-hexuloisomerase; *pgi*, glucose 6-phosphate isomerase; *gndA*, 6-phosphogluconate dehydrogenase; *zwf*, glucose 6-phosphate dehydrogenase.

Thus, these complete or partial plasmids were named pShoR14 and with a numerical suffix in descending order according to size: pShoR14-1 (21,512 bp; accession no. JAGHKT020000121), pShoR14-2 (possibly partial; 16,214 bp; accession no. JAGHKT020000027), pShoR14-3 (4439 bp; accession no. JAGHKT020000046), pShoR14-4 (3836 bp; accession no. JAGHKT020000049), pShoR14-5 (likely partial; 3025 bp; accession no. JAGHKT020000053), pShoR14-6 (2508 bp; accession no. JAGHKT020000058), and lastly, pShoR14-7 (2463 bp; accession no. JAGHKT020000059). Linear maps of these plasmids are shown in Figure 6.

The largest of the *S. hominis* ShoR14 plasmid contigs, pShoR14-1 (21,512 bp) contained a RepA_N replicase domain belonging to the *rep39* family and was almost identical to the plasmid “unnamed 1” from *S. hominis* FDAARGOS_136 (22,512 bp; accession no. CP014103), which we designated as pFDAARGOS_136-1. Both pShoR14-1 and pFDAARGOS_136-1 harbor the *qacAR* genes that confer resistance to quaternary ammonium compounds which are used as antiseptics or biocides in healthcare settings to control infection. Both plasmids are also potentially mobilizable due to the carriage of the *mobA/L* gene of the MOBQ family that encodes for a relaxase. MOBQ relaxases have been reported in both Gram-positive and Gram-negative plasmids such as the *S. aureus* plasmid pSK41 and the *Escherichia coli* plasmid RSF1010 [42]. Interestingly, both pShoR14-1 and pFDAARGOS_136-1 harbor several carbohydrate-metabolism-related genes including genes encoding for glucose 6-phosphate dehydrogenase (*zwf*) and glucose 6-phosphate isomerase (*pgi*) that are involved in central metabolism and this cluster of seven genes is flanked by IS*257* in a composite transposon-like structure. However, no direct repeats were identified flanking the two IS*257* copies. The possible function of this cluster of carbohydrate-metabolism-related genes is unknown and it is hard to imagine that enzymes which are involved in central carbohydrate metabolism, such as glucose 6-phosphate dehydrogenase and glucose 6-phophate isomerase, would be encoded on an accessory genetic element such as a plasmid. Indeed, a search of the genomes of *S. hominis* ShoR14 and FDAARGOS_136 showed that copies of these genes are located in their respective chromosomes, inferring the likelihood that these plasmid-encoded genes perform a different function as compared to their usual chromosomal counterparts.

The 4439 bp contig_46 designated plasmid pShoR14-3 contains the plasmid replication gene *repC*, which belongs to the *rep7a* family of replicases possessing a Rep_trans conserved domain. Additionally, it carries the tetracycline resistance gene, *tetK*. Plasmid-mediated tetracycline resistance in staphylococci is commonly associated with plasmids of the *rep7* family [43]. pShoR14-3 also carries a potential mobilization gene designated *mob/pre*. The pShoR14-3 plasmid is nearly identical (99.9% nucleotide sequence identity) to the well-characterized *S. aureus* mobilizable plasmid pT181 (4440 bp; accession number CP001783.1) [44].

The 3836 bp plasmid designated pShoR14-4 harbors the *repI* replication initiation gene also belonging to the *rep7a* family with a Rep_trans conserved domain. However, despite both replicases being of the same family, the pShoR14-4-encoded RepI only shared 77% amino acid sequence identity with the pShoR14-3-encoded RepC. This plasmid also contains the chloramphenicol acetyltransferase (*cat*) gene that confers resistance to chloramphenicol. Similar to pShoR14-3, the pShoR14-4 plasmid carries a *mob/pre* gene that belongs to the pMV158 superfamily relaxases which are widely distributed among Gram-positive and Gram-negative bacteria [42]. The closest homologues for pShoR14-4 are two unpublished and unnamed plasmids, one of which was from the *S. aureus* strain UP_1500 (99.83% sequence identity with 99% coverage to the 3785 bp plasmid with accession no. CP047814) and the other from the *Staphylococcus pseudintermedius* strain 081661 (95% sequence identity with 99% coverage to the 3785 bp plasmid with accession no. CP016074.1). Additionally, BLASTN analysis revealed a degree of similarity between pShoR14-4 and other large enterococcal plasmids with convergence of 60% or less and sequence identity of more than 80%. Two of these larger plasmids, namely *Enterococcus faecalis* plasmid pRE25, a large multi-resistance conjugative plasmid (50,237 bp; accession no. X92945.2) [45] and *E. faecalis* plasmid 4 (55,052 bp; accession no. LR962780.1), indicate the possibility of cointegration of similar small chloramphenicol resistance plasmids within larger plasmids. 

**Figure 7:**
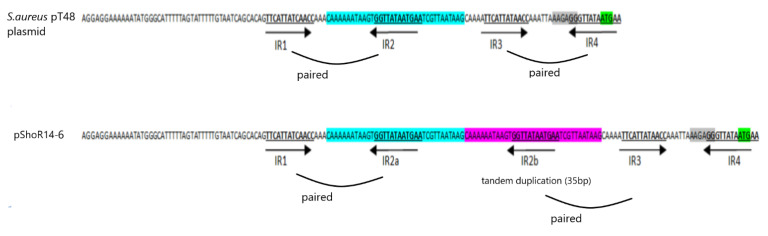
Regulatory sequence of the constitutively expressed *ermC* gene carried on pShoR14-6 described in this study and comparison with regulatory sequence of inducibly expressed *ermC* gene of *S. aureus* pT48 (accession no. NC_001395). The duplicated sequence comprising IR2b is highlighted in purple. The inverted repeats (IR1 to IR4) are indicated by arrows. The *ermC* start codon and e*rmC*-associated ribosomal binding site are highlighted with green and grey, respectively.

Another potential plasmid contig_58 (1759 bp) carrying *repL* and *ermC* genes was identified in the WGS data, and this contig was circularized by Sanger sequencing to obtain the entire plasmid sequence designated pShoR14-6 with a final size of 2508 bp. This plasmid showed 100% nucleotide sequence identity to the *S. hominis* strain Sho-115Lar plasmid (2473 bp; accession number NZ_MH423313) as well as to many other staphylococcal *ermC* plasmids. Its replication gene (*repL)* belongs to the *rep*10 family with a RepL conserved domain. No mobilization genes were found in this plasmid. The *ermC* gene is common among staphylococci and is mainly found in small 2.5 kb plasmids [46,47,48]. The *ermC* encodes a ribosomal RNA adenine N-6-methyltransferase that mediates resistance toward macrolides, lincosamides, and streptogramin B (MLS_B_) antibiotics and this resistance could be inducible (iMLS_B_) or constitutive (cMLS_B_). The inducible expression of the *ermC* gene is regulated by the *ermC* leader peptide coding sequence which encodes a small peptide and four inverted repetitive sequences (IR1, IR2, IR3, and IR4) that are capable of forming secondary structures which attenuate *ermC* translation [49,50]. Structural variations (deletion mutations, tandem duplications, or point mutations) in this *ermC* regulatory region can interfere with the regulatory mechanism, thereby leading to high expression of the *ermC* gene and constitutive resistance to macrolides and lincosamides [17,49,50]. The *S. hominis* ShoR14 strain exhibited the cMLS_B_ phenotype (i.e., constitutive) and sequence analysis of the *ermC* leader region in pShoR14-6 indicated that this is possibly due to duplication of a 35 bp segment comprising the inverted repeated sequence IR2b. A similar observation has previously been reported [50]. This duplication likely results in the pairing of IR1:IR2a and IR2b:IR3 leaving IR4 unpaired and thus, accessible to the ribosome (Figure 7). Accessibility of IR4 is essential to enable *ermC* translation, as this inverted sequence contains the *ermC* start codon and e*rmC*-associated ribosomal binding site. A study conducted by Szemraj et al. [7] indicated that cMLS_B_ was the predominant resistance type among various staphylococcal isolates. However, in an earlier study, Gatermann and co-workers found that the *ermC* gene was predominant among CoNS and constitutively expressed except for *S. hominis* subsp. *hominis* (*Shh*), which showed inducible *ermC* expression [51]. Another study also reported that the iMLS_B_ is a common resistance phenotype present in *S. hominis* [52].

Another putative plasmid contig of 2463 bp was found and designated pShoR14-7. Its replication initiator belongs to the *rep*21 family with a Rep_1 conserved domain. No resistance and mobilization genes were detected on this plasmid. However, BLASTP analysis showed that the pShoR14-7 replication initiator had a degree of similarity (55% identity and 96% coverage) with a replicative relaxase initiator previously found in *S. aureus* pUB110 plasmid (4548 bp; accession no. NC_001384.1). Several replication initiators of the Rep_1 family have been shown to function as mobilization relaxases in addition to their replication function [53]. 

In addition, another two possible plasmid contigs, i.e., pShoR14-5 (3025 bp) and pShoR14-2 (16,214 bp), were detected but these were likely to be partial sequences, as was mentioned earlier. The pShoR14-2 partial plasmid (contig_27) revealed a *repA* gene belonging to the *rep20* family with a RepA_N conserved domain as well as a recombinase-encoding gene (*sin*) and a copy of IS*257*. The presence of *sin* and especially IS*257* could explain the partial sequence that was obtained for pShoR14-2 as these elements play a role in genomic rearrangements that cannot be resolved using short-read WGS data. No significant homology for pShoR14-2 was found in the NCBI database. It has been reported that a majority of large staphylococcal plasmids (>20 kb) utilize replicases with a RepA_N conserved domain and this type of replicases is also widespread among large plasmids of other Firmicutes such as enterococci [54,55]. As for the partial pShoR14-5 plasmid (contig_53), it harbors the *repA* gene encoding a *rep19b* family replicase of the RepA_N conserved domain. No mobilization/conjugation and resistance genes were found in this partial sequence but its close homolog, plasmid p112250134 of the *S. aureus* strain 111250134 (34,958 bp; accession no. CP045443.1), carries a *mobA/L* mobilization gene along with resistance genes for cadmium, antiseptics (*qacA*), as well as penicillin (*blaZ*). pShoR14-5 shared >98% nucleotide sequence identity with p112250134 over a 3025 bp region (nts. 14,709–17,731; or coverage of 9%). However, a BLAST search of the other regions of p112250314 with the rest of the assembled contigs of ShoR14 did not lead to any conclusive results.

## 3. Conclusions

This study, which is the first report of the genome sequence of a multidrug-resistant, methicillin-resistant *S. hominis* strain ShoR14 from Malaysia, showed the diversity of genomic islands, prophages, and plasmids in ShoR14 and related *S. hominis* isolates. A novel variant of the SCC*mec* type VIII element was presented based on the complete genome sequence of the closely related *S. hominis* FDAARGOS_136. *S. hominis* ShoR14 likely harbors a similar SCC*mec* element, but this was unverifiable due to its spread over multiple contigs of the assembled short-read sequences. The possible presence of a SCC*fus* element in ShoR14 was similarly unvalidated. Resistance genes for tetracycline (*tetK*), chloramphenicol (*catA7*), macrolides, lincosamides, and streptogramin B (*ermC*), as well as antiseptics (*qacA*), were located on putative plasmids. Duplication of a 35 bp fragment in the *ermC* leader peptide region of pShoR14-6 likely led to the constitutive expression of *ermC* and the cMLS_B_ phenotype of ShoR14. The presence of resistance genes in mobile elements may lead to the emergence and spread of resistance in *S. hominis* and related staphylococcal strains in the hospital and other healthcare settings. Moreover, the genome sequence of ShoR14 indicated that the strain harbors virulence genes that facilitate adherence to host cells as well as immune evasion, allowing persistence and disease initiation, and thus warrants continual vigilance.

## 4. Materials and Methods

### 4.1. Bacterial Isolate Information 

The *S. hominis* ShoR14 was isolated from the blood culture of 70-year-old female patient, in 2016 from Hospital Sultanah Nur Zahirah (HSNZ), the main public tertiary hospital in the state of Terengganu, Malaysia, with the approval of the Medical Research and Ethics Committee, Ministry of Health Malaysia under the National Medical Research Registry Protocol No. NMRR-15-2369-28130 (IIR).

### 4.2. Antimicrobial Susceptibility and Disc Induction Test 

The *S. hominis* susceptibility profile was carried out against 26 antibiotics belonging to 18 antimicrobial classes as previously described [56]. The in vitro antimicrobial susceptibility data was interpreted based on the Clinical and Laboratory Standards Institute (CLSI) and the European Committee on Antimicrobial Susceptibility Testing (EUCAST) standards [57,58] (Supplementary Table S3). MLS_B_ resistance phenotype, i.e., inducible (iMLS_B_), constitutive (cMLS_B_), or clindamycin-susceptible and macrolide–streptogramin-B-resistant (MS) was determined using the D-test [46]. 

### 4.3. Whole Genome Sequencing, De Novo Assembly and Annotation

Short-read sequencing was performed on an Illumina HiSeq-PE150 high-throughput sequencer platform with paired-end sequencing strategy by a commercial service provider (Novogene Co., Ltd., Singapore). *De novo* assembly of the Illumina short reads was performed using Unicycler (v0.4.8) (https://github.com/rrwick/Unicycler assessed on 1 March 2021) [59]. The assembled draft genome was annotated using the PATRIC RASTtk-enabled Genome Annotation Service [60].

### 4.4. In Silico Molecular Typing

Identification of isolate sequence type (ST) was performed at PubMLST (https://pubmlst.org/ accessed on 1 June 2022) [61] and SCC*mec* element type was determined using SCC*mec*Finder available at the Centre for Genomic Epidemiology (https://cge.cbs.dtu.dk/services/ accessed on 1 June 2022). 

### 4.5. Bioinformatics

Comprehensive Antibiotic Resistance Database (CARD) [62] was used to identify resistance genes. Virulence factors were identified using the Virulence Factors Database (VFDB) [63,64]. Prophage regions were identified using the Phage Search Tool Enhanced release [65,66] and IslandViewer was used to predict the genomic islands (GIs) [67]. Comparative genome analysis was carried out using the NCBI BLAST+ tool kit available from (ftp://ftp.ncbi.nlm.nih.gov/blast/executables/blast+/LATEST/) then visualized by EasyFig 2.1 (http://mjsull.github.io/Easyfig/ accessed on 1 March 2021) [68] and also by CGView [69] at Proksee (https://proksee.ca/ accessed on 1 June 2022).

### 4.6. Plasmid Identification and Gap Closure

Initially, Bandage visualization program version 0.8.1 (https://rrwick.github.io/Bandage/ accessed on 1 March 2021) [70] and PlasmidFinder software version 2.1 available at the Center for Genomic Epidemiology database (https://cge.food.dtu.dk/services/PlasmidFinder/ accessed on 1 March 2021) [71] were used to obtain the potential plasmid contigs. Then, the complete/partial plasmid sequences were further determined using BLASTN search. A contig was considered a complete plasmid if the BLASTN results showed the best match with an entire reference plasmid, otherwise it was binned as a partial plasmid. The partial plasmid sequences were validated by PCR by designing primers directed outwards at the contig ends. PCR products (if obtained) were then sequenced by Sanger dideoxy sequencing. The primers used in this study for gap closure are listed in Supplementary Table S4.

### 4.7. Phylogenetic Analysis

The phylogenetic tree was constructed from the assembled ShoR14 genome along with 52 other assembled *S. hominis* strains available on GenBank (Supplementary Table S1) which were mainly selected based on the genome completeness and the quality metrics of the assemblies. The multiple sequence alignments of the core genome sequences of these strains were conducted using Roary (https://sanger-pathogens.github.io/Roary/ accessed on 1 March 2021) with core genomes identified using the criteria of amino acid sequence identities of >95% and presence in 99% of sampled genomes [72]. The derived core genome alignments were then used to infer maximum-likelihood (ML) trees using FastTree with 100 bootstraps under the GTR time-reversible model [73,74]. The resulting phylogenetic tree was visualized using iTOL v5 (https://itol.embl.de/ accessed on 1 June 2022) [75].

## Figures and Tables

**Table 1:** Identification of genes encoding for antimicrobial resistance from the genome sequence of *Staphylococcus hominis* ShoR14.

Antimicrobial Class	Resistance Phenotype	Resistance Gene	Mechanism of Resistance	Location of the Resistance Gene
β-lactams	Penicillin	*blaZ*	Antibiotic inactivation enzyme	Chromosomal
	Cefoxitin, oxacillin	*mecA, mecR1, mecI*	Antibiotic target alteration	Chromosomal
Fluoroquinolones	Ciprofloxacin, moxifloxacin	*norA*	Efflux pump conferring antibiotic resistance	Chromosomal
Macrolides	Erythromycin	*ermC*	Antibiotic target alteration	Plasmid
Lincosamides	Clindamycin			
Aminoglycosides	Gentamicin	*aac(6′)-aph(2″)*; *ant(4′)-Ib*; *aadD*	Antibiotic inactivation enzyme	Chromosomal
Folate inhibitors	Co-trimoxazole	*sul4, dfrC*	Antibiotic target replacement	Chromosomal
Fusidanes	Fusidic acid	*fusC*	Antibiotic target alteration	Chromosomal (SCC element)
Tetracyclines	Tetracycline, doxycycline	*tetK*	Efflux pump conferring antibiotic resistance	Plasmid
Phenicols	Chloramphenicol	*catA7*	Antibiotic inactivation enzyme	Plasmid
Monoxycarbolic acids	Mupirocin	*mupA*	Antibiotic target alteration	Chromosomal

**Table 2:** Genes encoding virulence factors identified from the *S. hominis* ShoR14 genome sequence.

Virulence Factor Classes	Gene	Product
Adherence	*atl*	Autolysin
	*ebp*	Elastin binding protein
Exoenzyme	*lip*	Lipase
	*nuc*	Thermonuclease
Immune modulation/evasion	*orf01763, orf02129, orf02130, orf02131, orf02132, orf02135, orf02139, orf02141*	Capsule biosynthesis proteins
	*capB*, *capC*	Polyglutamic acid capsule

**Table 3:** Characteristics of the transposon-like element Tn*7546* and its target site duplication in *S. hominis* and other bacterial species where the element is detected.

Bacterial Species	Position of Tn*7546*	Size of Tn*7546* (bp)	Length of Target Site Duplication (bp)	Target Site Duplication Sequence	Accession Number
*Staphylococcus hominis* ShoR14	Contig_9: 23,335..19,532	3789	8	AAAATAAG	JAGHKT010000009.1
*Staphylococcus hominis* FDAARGOS_136	1,838,641..1,842,445	3789	8	AAAATAAG	CP014107
*Staphylococcus hominis* FDAARGOS_661	958,241..962,044	3789	8	AAAATAAG	CP054550
*Staphylococcus hominis* 19A	2,187,352..2,183,549	3789	8	AAAATAAG	CP031277
*Staphylococcus hominis* C34847	1,015,480..1,009,762	3789	8	AAAATAAG	CP014567
*Staphylococcus epidermidis* NW32	42,074..45,879	3790	8	AAAATAAG	KT726221
*Lysinibacillus fusiformis* RB-21	271,488..267,683	3790	8	TATTAAAC	CP010820
*Lysinibacillus fusiformis* NEB1292	1,557,552..1,561,356	3791	7	ATTAAAC	CP070490
*Lysinibacillus spaericus* IAB59	4,592,034..4,595,839	3790	8	GTTTAATA	CP071741
*Salinococcus halodurans* H3B36	6,428..10,233 2,758,300..2,762,105	3790 3790	8 8	TTTAAAAT TTTAAAAT	CP011366
*Rothia aeria* LPB0401	2,602,067..2,605,872	3790	8	TTGTTAAG	CP079819
*Granulicatella elegans* FDAARGOS_1559	256,390..260,195	3790	8	ATTTTTAT	CP085953
*Haemophilus parainfluenzae* M1C42_1	1,337,751..1,341,555	3790	8	AAAATTAT	CP063117
*Streptococcus agalactiae* 515	1,870,022..1,873,812	3775	8	TTTAAATT	CP051004
*Streptococcus dysgalactiae subsp. equisimilis* NCTC7136	1,516,598..1,520,403	3790	8	AAAAAATC	LS483413
*Streptococcus mitis* B6	838,468..842,273	3790	8	TTATTTAT	FN568063
*Streptococcus pneumoniae* 2245STDY5775520	1,015,340..1,019,131	3776	8	GAAATATA	LR216027

## Data Availability

The draft genome sequence of *Staphylococcus hominis* ShoR14 has been deposited in GenBank under the accession no. JAGHKT000000000.

## Supplementary Materials

The following supporting information can be downloaded at: https://www.mdpi.com/article/10.3390/pathogens11121406/s1, Figure S1: Comparative linear maps of SCC*fusC* (accession no. KF527883) with SCC*mec* types VIII(4A) (accession no. FJ390057) and V(5 and 5C2) (accession no. AB505629) with mapping of the *S. hominis* ShoR14 contigs to these elements; Figure S2: Comparative genomic island 3 (GI-3) of S. hominis ShoR14 predicted by IslandViewer 4 to be 11,216 bp in length in the FDAARGOS_136 genome; Table S1: List of *Staphylococcus hominis* strains used to construct phylogenetic tree; Table S2: Genomic islands (GIs) of *Staphylococcus hominis* strain FDAARGOS_136 (CP014107.1); Table S3: Antimicrobial concentration and interpretative values for ShoR14; Table S4: List of primers used in this study for gap closure.
