# Supplementary material for: Comparative Genomic Analysis of a Multidrug-Resistant Staphylococcus hominis ShoR14 Clinical Isolate from Terengganu, Malaysia, Led to the Discovery of Novel Mobile Genetic Elements
Source: Pathogens. 2022 Nov 23;11(12):1406. doi: 10.3390/pathogens11121406 (PMC9782665; doi:10.3390/pathogens11121406)
Supplement: Supplementary file 1 [file pathogens-11-01406-s001.zip › Supplementary Figures S1 & S2.pdf]

Supplementary materials:

Comparative genomic analysis of a multidrug-resistant *Staphylococcus hominis* ShoR14 clinical isolate from Terengganu, Malaysia, led to the discovery of novel mobile genetic elements

Esra'a I. Al-Trad [1], Ainal Mardziah Che Hamzah [2], Suat Moi Puah [3], Kek Heng Chua [3], Stephen M. Kwong [4], Chew Chieng Yeo [1,*] and Ching Hoong Chew [2,*]

Supplementary Figure S1

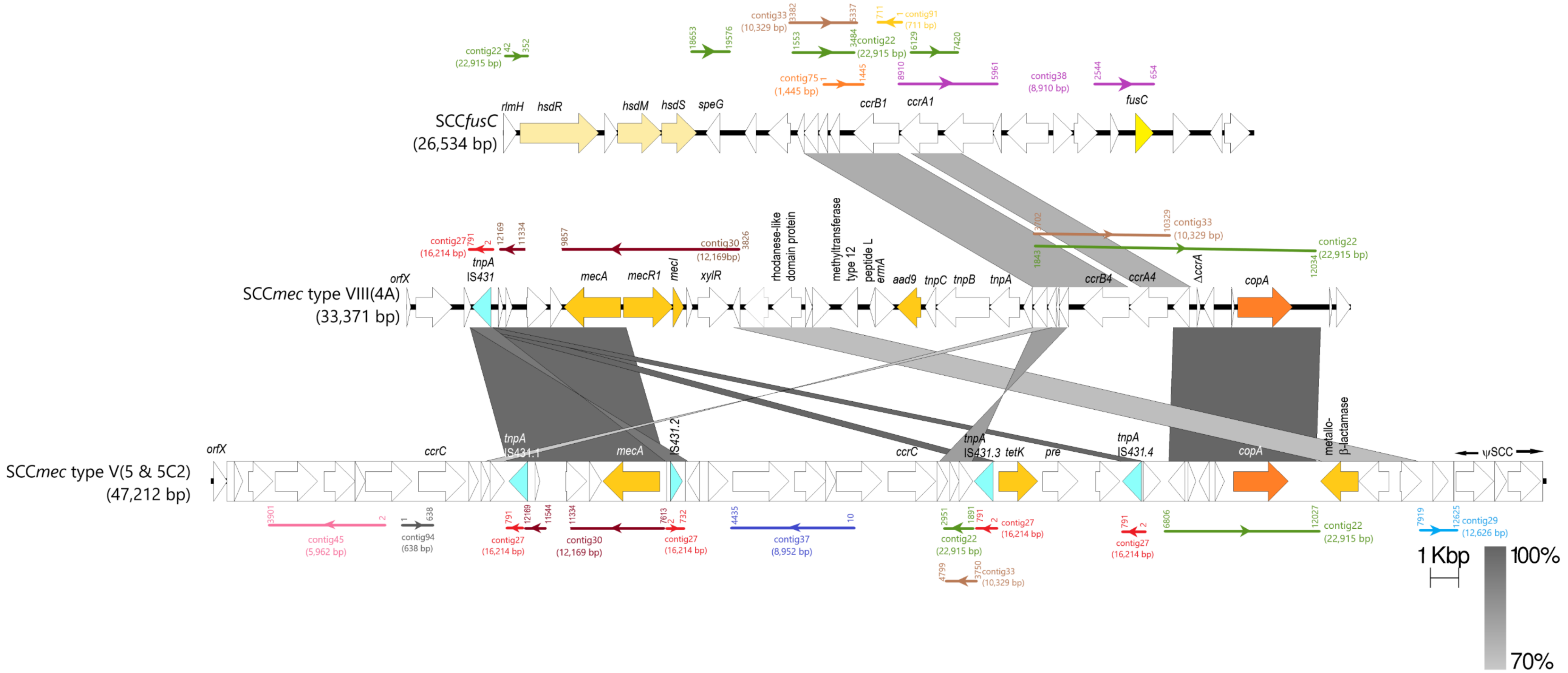

Supplementary Figure S1. Comparative linear maps of SCC*fusC* (accession no. KF527883) with SCC*mec* types VIII(4A) (accession no. FJ390057) and V(5 & 5C2) (accession no. AB505629) with mapping of the *S. hominis* ShoR14 contigs to these elements. The ShoR14 contigs were indicated as horizontal lines above the SCC*fusC* and SCC*mec* type VIII(4A) maps and below the SCC*mec* type V(5 & 5C2) map, and were colored accordingly and labeled with their respective nucleotide coordinates from which they shared sequence identities of >90% with the SCC elements. Arrows on the contigs depict the direction of their homologies. For the linear maps, sky blue arrows are transposases of IS elements, golden arrows are antibiotic resistance genes, yellow arrow labelled as *fusC* is the fusidic acid resistance gene, orange arrows depict metal resistance genes.

Supplementary Figure S2

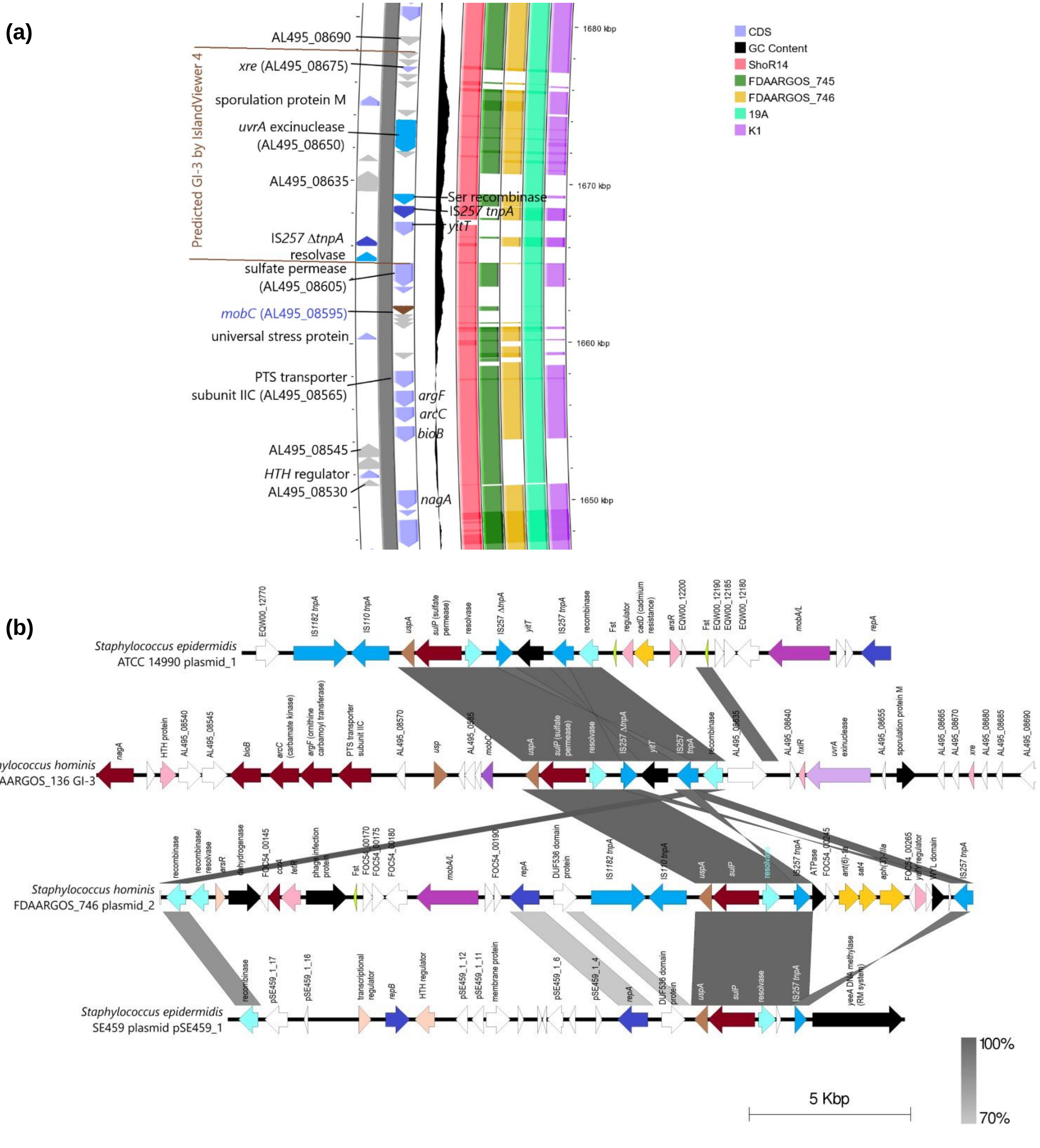

Supplementary Figure S2. Comparative genomic island-3 (GI-3) of *S. hominis* ShoR14 predicted by IslandViewer 4 to be 11,216 bp in length in the FDAARGOS_136 genome. (**a**) Closeup of the CGView comparison of the genomes of *S. hominis* FDAARGOS_136 (outer two rings) with *S. hominis* ShoR14 (fourth ring from outside indicated in pink), *S. hominis* FDAARGOS_745 (fifth ring in dark green), *S. hominis* FDAARGOS_746 (sixth ring in gold), *S. hominis* 19A (seventh ring in apple green) and *S. hominis* K1 (innermost ring in purple) as depicted in Figure 2 but showing the region surrounding the genomic island GI-3 predicted by IslandViewer 4 (indicated as flanked by brown horizontal lines) from the complete genome sequence of FDAARGOS_136. (**b**) Comparative linear maps of the putative GI-3 shown in (a) with several staphylococcal plasmids showing the region of homology (>70% nucleotide sequence identities) between the GI-3-encoded *uspA* gene encoding a universal stress protein (AL495_08575; shown as light brown arrow) and either the serine recombinase protein (AL495_08630) or the IS*257* downstream of the resolvase (AL495_08600). The plasmids depicted are *Staphylococcus epidermidis* ATCC 14990 plasmid_1 (accession no. CP035289), *S. hominis* FDAARGOS_746 plasmid_2 (accession no. CP046305), and *S. epidermidis* SE459 plasmid pSE459_1 (accession no. MW364982). IS-encoded transposases are indicated as blue arrows, resolvases and recombinases are shown as sky blue arrows, plasmid replicase genes are dark blue arrows, purple arrows are plasmid mobilization-related genes, pink arrows are putative transcriptional regulators, brown and black arrows are genes with known functions or domains, respectively, while light green arrows are the Fst toxin of the type I toxin-antitoxin system and finally white arrows are hypothetical CDS.

Please refer Excel for supplementary tables,

Table S1: List of *Staphylococcus hominis* strains used to construct phylogenetic tree.

Table S2: Genomic islands (GIs) of *Staphylococcus hominis* strain FDAARGOS_136 (CP014107.1).

Table S3: Antimicrobial concentration and interpretative values for ShoR14.

Table S4: List of primers used in this study for gap closure.
